# The Promotion of Non-Communicable Disease Screening in Gurage Zone, Ethiopia: A Mixed-Method Study

**DOI:** 10.3390/diseases12110294

**Published:** 2024-11-17

**Authors:** Heldana Debebe, Bezawit Ketema, Sophie Sarah Rossner, Sarah Negash, Adamu Addissie, Mirgissa Kaba, Mulugeta Tamire, Eva Johanna Kantelhardt

**Affiliations:** 1 Global Health Working Group, Martin Luther University, 06097 Halle, Germanyeva.kantelhardt@uk-halle.de (E.J.K.); 2 Institute for Medical Epidemiology, Biostatistics and Informatics, Martin-Luther-University, 06097 Halle, Germany; 3 School of Public Health, College of Health Sciences, Addis Ababa University, Addis Ababa 9086, Ethiopia

**Keywords:** health communication, screening, satisfaction, non-communicable disease, behavioral change, primary healthcare facility

## Abstract

Background: Despite the high non-communicable disease (NCD) mortality in Ethiopia, NCD screening in the country remains suboptimal. This study assessed the health communication process and materials designed to promote NCD screening among adult primary healthcare facility attendants in the Gurage Zone, Ethiopia. Methods: A parallel mixed-methods approach was employed. Seven health communication materials were evaluated using the Modified Clear Communication Index Score by the Center for Disease Control and Prevention (CDC). Seven key informants who were involved in the production were interviewed to explore the process. Users’ satisfaction with the materials was assessed through a cross-sectional survey (N = 412). We used multivariable logistic regression with SPSS version 27 software to determine the factors associated with users’ satisfaction. Thematic analysis was applied for the qualitative data analysis using Opencode 4.03 software. Results: Qualitative interviews indicated that the production process relied on evidence, involved stakeholders, and included pretesting. The CDC index score revealed that five of the seven materials were considered clear and user-friendly, whereas two required improvement. Overall users’ satisfaction with the material was high with a mean score of 22.10 (SD ± 2.34; min: 14, max: 25). Age and educational status had significant positive association with users’ satisfaction. Conclusions: Developing health communication materials on promoting NCD screening based on evidence, stakeholders’ input, and pretesting can lead to good quality material and user satisfaction. We recommend future research works to measure changes in NCD screening service uptake as a result of using the health communication materials considered in this study.

## 1. Introduction

Globally, non-communicable diseases (NCDs) are the primary causes of death [1]. Of all global NCD deaths, 77% are in low- and middle-income countries [2]. The main types of NCDs are cardiovascular diseases, cancers, chronic respiratory diseases, and diabetes [2]. In Ethiopia, every hour, an estimated 25 Ethiopians are dying of NCDs, with an estimated rate of 554 (95% UI: 502–605) per 100k population [3]. In Ethiopia, disability-adjusted life years (DALYs) due to NCDs have increased from 20% in 1990 to 69% in 2015 [4]. However, though the shift in NCD burden has been the most rapid, Ethiopia is among the countries least prepared for this transition [5]; NCD screening service uptake in the country is still very low [6,7,8], and NCD communication, in particular, and health communication, in general, did not receive as much priority as other health-related activities in the country [9].

International entities such as the World Health Organization (WHO) and the United Nations (UN) have identified the prevention and control of NCDs as an increasingly important discussion item on the global health agenda [10,11]. In congruence with global commitments, the Ethiopian national guideline on major NCDs aims to reduce their increasing burden in Ethiopia through primary (vaccinations), secondary (screening), and tertiary (diagnosis, treatment, and care) levels of prevention [12]. In addition, the Ethiopian national strategic action plan for the prevention and control of major NCDs aimed to increase public awareness on NCDs by conducting social and behavioral change communication through print and electronic media [13]. Furthermore, the Ethiopian National Health Promotion and Communication Strategy Framework emphasized that without increased public awareness and behavior change, health improvements in the country would not be achievable [14].

Thus, efforts towards NCD prevention and control in Ethiopia have encompassed the development of health communication materials to promote NCD screening [15,16,17]. The NCD working group at the School of Public Health, Addis Ababa University, exemplifies this approach of promoting NCD screening, creating seven different materials encompassing an audiovisual presentation and six printed pieces (leaflets, posters, and a billboard). The main message of these materials was about NCD screening. The targeted NCD screening services in this study were clinical breast examination, cervical cancer screening, blood pressure measurement, and blood glucose measurement. The audiovisual component features healthcare professionals encouraging regular screening, along with testimonials from survivors emphasizing the benefits of early detection. Posters stress the ease of NCD screening, the often asymptomatic nature of these diseases, and the importance of a healthy lifestyle. Leaflets give definitions of NCDs, their risk factors, potential complications, and preventative measures. The billboard visually shows the targeted NCD screening procedures (Materials S1).

However, most behavioral change communication processes and materials developed to communicate health issues in Ethiopia are not evaluated or not to satisfactory quality [18,19]. Thus, this study assessed the production process, materials’ quality, and the user satisfaction of the NCD screening communication materials developed by the School of Public Health, Addis Ababa University, for adult primary healthcare facility (PHF) attendees in Gurage Zone, Ethiopia. Thus, policymakers in Ethiopia and similar settings can learn from the process and adopt the materials for promoting NCD screening for the prevention and control of NCDs.

## 2. Materials and Methods

### 2.1. Study Design, Area, and Period

A facility-based parallel mixed-method approach was used in this study. A qualitative descriptive program evaluation study approach was applied to explore the material production process. To measure the level of satisfaction with the audiovisual material among PHF attendants, a facility-based cross-sectional study design was used. This study took place at two PHFs in Gurage Zone, Ethiopia. These are the sites where the health communication materials developed by the NCD working group at Addis Ababa University’s School of Public Health were disseminated. Gurage Zone is located in the regional state of Central Ethiopia, which is the third most populous region in the country with an estimated population of 9.3 million in 2024 [20]. This study’s data were collected from 1 to 30 January 2022.

### 2.2. Sample Size and Sampling Procedures

For the quantitative data, a sample size was determined using a single population proportion formula. With the assumption that there are no prior national or local data used in a similar setting on satisfaction with the health communication materials among adult PHF attendants, a sample proportion of 50% was used to maximize the sample size. Considering a 95% confidence interval, a 5% margin of error, and a 10% anticipated non-response rate, the sample size (N) was calculated at 422.

Participants were selected using the systematic random sampling method among adults attending the two PHFs where the health communication materials considered in this study were disseminated. At those PHFs, the audiovisual was being played at the waiting areas, posters were posted outside the service room walls, billboard was placed inside the facilities at the entrance next to the internal navigation sign, and leaflets were being given by the healthcare professionals for all attendants in all service rooms. Ten days before the actual data collection, clients’ flow rate to each of the facilities was calculated, and no difference in the flow rate for the next one month of data collection was assumed. The flow rate was 60 adults per facility per day. The sample size from each facility/date of data collection (211/30 = 7) was the sample size from each facility per day. The sample size interval was computed as (60/7 = 8). Therefore, the other eight adults visiting each of those PHFs were approached and interviewed.

For the qualitative data, seven key informants who participated in the production, evaluation, or dissemination of materials were selected using the critical-case purposive sampling method. Participant recruitment was stopped at the saturation level. Moreover, all seven health communication materials on NCD screening produced by the NCD working group at the School of Public Health, Addis Ababa University, were evaluated in this study.

### 2.3. Measurements and Data Collection

Quantitative data were collected at the exit gate of those selected PHFs once respondents finished their stay at the facility. An interviewer-administered questionnaire, adapted from a satisfaction assessment literature, was used to collect the quantitative data [21]. A structured questionnaire was developed in the English language, translated into the local language (Amharic), and then translated back into English by language experts. The first draft questionnaire was pilot-tested and appropriate modifications were made.

The final version of the questionnaire was structured into three sections: socio-demographic characteristics; exposure to the materials; and satisfaction with the audiovisual materials. Satisfaction was measured with five items on a five-point Likert scale, with responses ranging from ‘very dissatisfied’ (1) to ‘very satisfied’ (5), with a minimum possible score of ‘5’ and a maximum possible score of ‘25’. Satisfaction items assessed the overall presentation/appearance, attractiveness, understandability, and appropriateness of the material in terms of culture, as well as its ability to offer new information (Table S1). Each of the satisfaction item scores were summed up and the mean satisfaction score was calculated. The mean value was used to dichotomize the satisfaction variable. Respondents who scored above the mean were considered satisfied with the material.

The qualitative data were collected using a semi-structured key informant interview guide to explore the production process of the health communication materials. Most of the qualitative interviews took place at the respondent’s office. Each interview took 30 min on average. The quality of the materials was evaluated using the modified CDC clear communication index score [22]. The modified index has a total of 13 items under 4 sections. The number of items scored depends on the type of material. The four sections are core, behavioral recommendations, numbers, and risk. The ‘core’ includes the main message, call to action, and language (Table S2). A four-day intensive training on quantitative and qualitative data collection was given for the data collectors prior to the actual data collection.

### 2.4. Data Processing and Analysis

The quantitative data were checked for completeness, coded, and entered into SPSS version 27 for analysis. Descriptive statistics were used to describe the frequency distribution, proportion, measures of central tendency, and dispersion. The association between single explanatory variables and dependent variables was examined through bivariable analysis by computing the odds ratio at a 95% confidence level. A multivariable logistic regression model was used to determine factors associated with the satisfaction and to control confounding variables. Crude and adjusted odds ratios with 95% confidence intervals were calculated. Six variables were included in multi-variable logistic regression analysis based on *p*-values less than 0.25 in bivariate analysis [23], the consideration of multi-collinearity, clinical significance, and the maximum number of variables that is sufficient to enter the model. For all statistical significance tests, independent and dependent variables were declared at *p* < 0.05.

Thematic analysis was used to understand the qualitative data and is presented with quotations. According to the CDC clear communication index score, the overall score is out of 100%. A material with a score of 90 or higher is considered good, easily understandable, and usable by the audience. A material with a score of 89 or lower is considered for improvement [22].

## 3. Results

### 3.1. Health Communication Material Production Process

#### 3.1.1. Characteristics of Key Informant Interview Participants

Seven purposely selected individuals gave a key informant interview in this study. These individuals took different roles in the production process of the health communication materials considered in this study. Key informants were aged 29 to 52 years, two of the seven participants were females, and they had a range of professions (e.g., one clinician, one musician, two producers, one graphic designer, one photographer, and one health communication expert) (Table 1).

#### 3.1.2. Bases for Developing the Materials: Evidence-Driven?

This study’s participants articulated that producing the health communication materials considered in this study was set to be a valuable response to the results of an earlier baseline study, which showed different factors associated with the low NCD screening practice among the target audience.

*…Before the start of the material development, there was a baseline study, and the result of the baseline study was presented to us. The study indicated different wrongly held beliefs for the low NCD screening behavior in the study area. The health communication materials we developed was aimed towards changing those beliefs…*

(KII 07)

*…According to the baseline study findings, most of our audiences believe that they are healthy, and that is why they fail to get screened… thus, through our materials we indicated that NCDs are asymptomatic at their early stage…*

(KII 01)

Key informants also emphasized the usage of an evidence-based media mix to increase the reach.

*…When the baseline study finding was presented to us, we came to know that some of our target audiences can’t read and write, then we decided to develop an audiovisual in addition to printed materials… For those who can’t read the printed materials, they can watch the video…*

(KII 03)

Key informants indicated that, in addition to the baseline study, they drew upon various guidelines and manuals during the development of these health communication materials.

*…When we developed these materials, we used the CDC guidelines, the USAID video-shooting guide, and the Ethiopian national health promotion and communication strategy…*

(KII 05)

#### 3.1.3. Strategies Used in Developing the Materials

Developers of these communication materials used different strategies to command attention, to clarify the message, to convey benefit, and to build trust among target audiences. The use of local language, including sign language translation in the audiovisual field, the linking of art and science, media mixing, the involvement of community representatives, and stakeholder engagement were some of the strategies mentioned by the key informants.

*…Our photographer first went to the field and took some photos that could reflect the target population. Then, individual actors in these materials designed following a dress code, age group and sex that best fits to the culture and topic…*

(KII 06)

*…We developed and produced a new music track for this purpose, and we used it as a background in audiovisual… that is to attract attention…*

(KII 02)

#### 3.1.4. Pretesting and Evaluation of the Materials

The key informants reflected that the materials underwent evaluations at different phases of the production process. The evaluations were concept-tested before the first draft, the stakeholders’ review after first draft, and field testing after the final draft. The evaluations resulted in the changing of wordings, slogans, colors, and signs.

*…Before we started the actual production, we first presented the storyline to the team and we revised it according to the feedback, then we developed our first draft… the draft materials were revised many times upon feedback from different stakeholders including community representatives; our final evaluation was made before we duplicated the materials, where we did the pretest in the community that resembled our target audience…*

(KII 05)

*…In a similar vein, a key informant who developed the music lyrics stated that, because of comments from various stakeholders, some parts of the music lyrics had been completely cut down and replaced with other words. Not only was that, but the addition of a female vocalist required to balance male and female participation and create a more engaging environment for both sexes…*

(KII 02)

The key informants emphasized several challenges that were faced during the production of the material. Finding real patients willing to participate in the video recording was the main difficulty. The team also encountered challenges in script development, with the aim of expressing cultural touches and using appropriate language for all four NCDs in common.

*…It was difficult to make the storyline based on all the four target NCDs in a single material. However, after having repeated discussion with the team, we managed to address all of the targeted NCDs by presenting them one by one in the audiovisual, by producing two different leaflets according to the disease type, and by focusing on the common features on the rest of the materials…*

(KII 05)

Moreover, the creative team leader acknowledged encountering various challenges related to COVID-19 restrictions, though they highlighted that those obstacles were successfully managed through dedicated efforts.

### 3.2. Quality of the Health Communication Materials: CDC Clear Communication Index Scores

The main message of the disseminated materials was about NCD screening. The majority of materials explained NCD risk factors and the benefits of adopting other NCD preventive behaviors.

In all the materials, the main messages and calls to action were presented in the active voice using audience-friendly language. On the two leaflets, the main message was written at the end or on the back of the material. On all other materials, the main message was written at the top, the beginning, or at front section. Calls to action, such as getting screened and reducing NCD risk factors, were consistently covered within the materials. Moreover, visuals and images reinforced the recommended behaviors. To enhance readability, some printed materials employed bulleted lists to break up the text. In addition, decimals, fractions, and percentages were avoided for clarity.

The CDC clear communication index score results revealed that five of the seven materials scored above 90%. This indicates they are good materials and have addressed most of the items that make materials easier to understand and use. However, the two leaflets scored below 89%, suggesting they have areas for improvement (Table 2).

### 3.3. Users’ Exposure and Satisfaction with the Health Communication Materials

#### 3.3.1. Socio-Demographic Characteristics of Survey Participants

Complete responses for the quantitative survey were obtained from 412 participants, with a 97.6% response rate. Most of the participants (176; 42.7%) were in the age range of 30–39 years, and a few (27; 6.6%) were between 60 and 69 years. More than half (238; 57.8%) of participants were females. Most of the participants (340; 82.8%) were married. The majority of the respondents (190; 46.1%) could not read or write. With regard to the respondents’ occupations, most of them (141; 34.2%) were farmers (Table 3).

#### 3.3.2. Exposure to the Health Communication Materials

**Figure 1:**
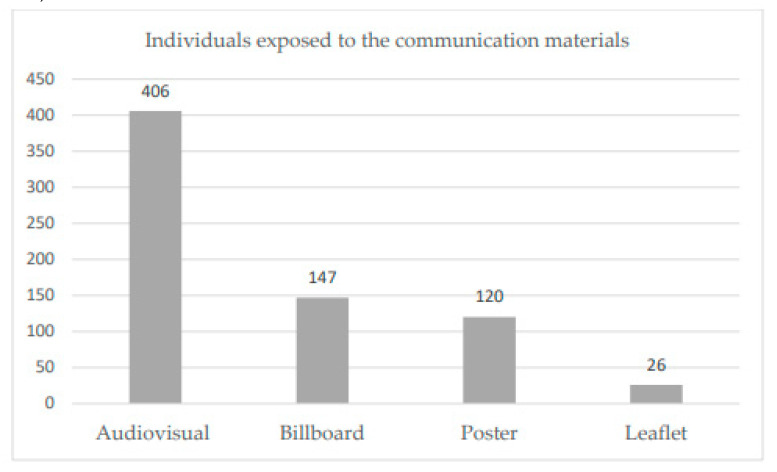
Number of individuals who were exposed to the health communication materials (N = 412).

After the completion of the visits to those facilities, exposure to the health communication materials differs among the 412 participants in this study. The majority (406; 98.5%) saw the audiovisual presentation during their visit to the PHFs. However, exposure to printed materials was lower; only 26 (6.3%) saw at least one of the two leaflets, while 147 (35.7%) saw the billboard, and 120 (29.1%) saw at least one of the three posters (Figure 1).

#### 3.3.3. Satisfaction with the Audiovisual

Satisfaction with the audiovisual was assessed among the 406 participants who had seen it. Participants found the presentation, attention-grabbing aspects, and ease of understanding to be the most satisfactory aspects, with mean scores of 4.50 (SD ± 0.54; min: 2, max: 5), 4.48 (SD ± 0.60; min: 2, max: 5), and 4.41 (SD ± 0.68; min: 1, max: 5), respectively. Scores for cultural appropriateness and providing new information were slightly lower at 4.37 (SD ± 0.69; min: 2, max: 5) and 4.33 (SD ± 0.76; min: 2, max: 5), respectively. Each satisfaction item score was summed up and gave observed values ranging from 14 to 25, and mean satisfaction score was computed as 22.10 (SD ± 2.34) (Table 4).

#### 3.3.4. Factors Associated with Satisfaction with the Audiovisual

From the multivariable logistic regression analysis, respondents’ age and educational status were significantly associated with satisfaction with the audiovisual material. The association showed that respondents aged 40–49 years were twice as likely to be satisfied with the audiovisual than respondents aged 30–39 years (AOR: 2.20, 95% CI: 1.33–3.63). Respondents who went through university/higher education were 4.4 times (AOR: 4.42, 95% CI: 1.36–14.40) more likely to be satisfied with the audiovisual material than those who cannot read and write (Table 5).

## 4. Discussion

This study evaluated the health communication process, materials disseminated, and users’ satisfaction on the health communication materials developed by the School of Public Health, Addis Ababa University, for increasing NCD screening service uptake among PHF attendants in Gurage Zone, Ethiopia.

After visiting the facilities where the study communication materials were distributed, participants showed varying levels of exposure to the materials. The vast majority (98.5%) saw the audiovisual presentation during their visit to the PHFs, while exposure to printed materials was considerably lower. This could suggest that the audiovisual material had the highest reach within the healthcare facilities. In line with this finding, a similar study conducted in Bangladesh, assessing the role of print materials and electronic media to improve cervical cancer screening, revealed that television was the best method of awareness creation [24]. This may be due to the fact that printed materials primarily reach literate individuals and exclude those with visual impairments, whereas the audiovisual format, displayed on a television screen, can effectively engage both literate and non-literate audiences. Moreover, individuals with visual impairments can benefit from the audio component, making the information more accessible. Evidences also indicated that most audiences preferred audiovisuals than print materials [25,26,27]. By the time they finished their visit to those PHFs, only 29% of the study participants reported seeing the posters targeted in this study. Similarly, following the poster campaign where four hundred posters for smoking prevention were placed in the streets of Geneva, only 36% of respondents reported seeing posters about smoking prevention [28]. This may indicate the possible lower reach of printed materials, especially posters, compared to audiovisuals.

In this study, the high mean scores across all audiovisual material satisfaction items (22.10 (SD ± 2.34; min: 14, max: 25)) suggest that target audiences are generally satisfied with the audiovisual material. As per a study conducted in Addis Ababa, Ethiopia, the received service has to meet or exceed the patients’ expectations for the patients to be satisfied [29]. Thus, our study findings could imply that audiovisual material has met or exceeded target audiences’ expectations.

The multivariable logistic regression report of this study showed that participants’ age and educational status have a positive association with satisfaction with the audiovisual material. Respondents who went through university/higher education were 4.4 times (AOR: 4.42, 95% CI: 1.36–14.40) more likely to be satisfied with audiovisual material than those who cannot read and write. A positive association between educational status and satisfaction was observed in studies measuring satisfaction with other healthcare services as well [30,31]. This may be due to the comparably greater extent of attention and understanding of respondents who went through university/higher education. Respondents who went through university/higher education are typically more knowledgeable about their medical condition and treatment options, making them more likely to have a better understanding of the care they received and thus be more satisfied with it. Moreover, respondents in the age range of 40–49 years were 2.2 times more likely to be satisfied with the audiovisual material than those who are in the age range of 30–39 years. This could be due to the fact that older people are more likely to pay more attention to NCDs than younger people. In other similar studies, a variety of socio-demographic characteristics, including educational status and age, were identified as factors influencing patient satisfaction, as these characteristics can provide insight into a patient’s expectations and experiences [21,32,33]. Another possible explanation for statistically significant satisfaction with the audiovisual material among those aged 40-49 years was that, in this study, compared to older age ranges, respondents in the age range of 40-49 years were mostly those who went through university/higher education, and acquiring higher education has showed to be significantly positively associated with the satisfaction.

In this study, the CDC clear communication index score result showed that, from the total of seven materials, on five of them, the main messages were written at the top, beginning, or on the front section of the materials. However, on the two leaflets, the main message was written at the end or at the back of the material. As per the CDC standard, when the main messages are written at the top or in first section of the material, audiences can find them more easily and quickly, and vice versa [22]. However, according to other similar studies conducted in Ethiopia, most of the evaluated health communication materials on cholera [18] and maternal and child health [19] were largely of poor quality and needed improvement due to a lack of pretesting, the requirement of assessments, and community involvement. However, in this study, the placement of the key message was the reason for low quality not the lack of assessment, pretesting, or community engagement.

As evidenced by the key informants of this study, there was a baseline assessment conducted prior to the material production to identify factors associated with NCD screening behavior, to characterize the target audience, and to understand the setting. Furthermore, this intervention package encompasses both printed and audiovisual channels to promote NCD screening among the target audience. Such evidence-driven health communication material production and media mixing have been recommended to ensure the take-up of the desired behavior in other literature studies as well [34,35,36]. Thus, policymakers in Ethiopia and similar settings can learn from the process and adopt the materials for promoting NCD screening in the prevention and control of NCDs.

### Limitations of the Study

As this study is a facility-based cross-sectional study, its findings might not be representative of the general population. Furthermore, this study was unable to assess whether those who reported being satisfied actually used the screening service. In addition, as respondents were exposed to the different communication materials in different ways, we only assessed satisfaction with the audio-visual material, which might not show satisfaction with printed materials.

## 5. Conclusions

This study assessed the health communication process and materials designed to promote NCD screening among adults who attended a primary healthcare facility in Gurage Zone, Ethiopia. The material production process was based on evidence, stakeholders’ input, and pretesting. Most of the materials were considered clear and user-friendly. There was higher end-user satisfaction with the materials considered in this study. Developing health communication materials based on evidence, stakeholders’ input, and pretesting can lead to good quality material and higher end-user satisfaction. Thus, we recommend future research works to measure the effectiveness of these health communication materials in increasing NCD screening service uptake.

## Figures and Tables

**Table 1:** Characteristics of key informant interview participants.

KII Participant	Age and Sex	Profession	Role in Material Production
01	38-year-old female	Internist and clinical oncologist	Content revision and edition
02	40-year-old male	Musician	Sound development
03	34-year-old male	Health professional, head of Woreda Health Office	Material review
04	52-year-old male	Producer	Audiovisual content development and production
05	45-year-old male	Producer	Print material content development
06	29-year-old female	Graphic designer	Graphic design and photography
07	40-year-old male	Health communication expert	Leader, content edition

**Table 2:** Quality health communication materials: CDC clear communication index score.

Materials	Core (Main Message, Call to Action, Language)	Behavioural Recommendations	Numbers	Risk	Total Score
Leaflet 1	4/6	2/2	2/2	2/2	83.3
Leaflet 2	4/6	2/2	NA	2/2	80.0
Billboard	6/6	1/1	NA	NA	100.0
Poster 1	6/6	1/1	NA	NA	100.0
Poster 2	6/6	1/1	NA	NA	100.0
Poster 3	6/6	1/1	NA	NA	100.0
Audiovisual	6/6	2/2	NA	2/2	100.0

**Table 3:** Socio-demographic characteristics.

Variables	Response	Frequency (N = 412)	Percentage (%)
Age in years	30–39	176	42.7
	40–49	147	35.7
	50–59	62	15
	60–69	27	6.6
Sex	Male	174	42.2
	Female	238	57.8
Religion	Orthodox	237	57.5
	Muslim	125	30.3
	Other *	50	12.1
Marital status	Single	41	10.0
	Married	340	82.8
	Divorced	10	2.4
	Widowed	21	5.1
Educational status	Cannot read and write	190	46.1
	Can read and write without primary school	24	5.8
	Primary school	80	19.4
	Secondary school	51	12.4
	Technical	11	2.7
	University/higher education	56	13.6
Occupation	Farmer	141	34.2
	Merchant	89	21.6
	Private employee	35	8.5
	Government employee	58	14.1
	Other **	89	21.6

* (Protestant, Catholic, or Adventist); ** (housewife, pensioner, or daily laborer).

**Table 4:** Level of satisfaction towards the audiovisual material (N = 406).

Satisfaction category	Minimum	Maximum	Mean	Standard Deviation
Presentation of the audiovisual	2	5	4.50	0.539
Attracting attention	2	5	4.48	0.595
Easiness to understand	1	5	4.41	0.679
Appropriateness of the audiovisual in terms of culture	2	5	4.37	0.690
Giving new/different information	2	5	4.33	0.761
Overall satisfaction towards the audiovisual material	14.00	25.00	22.10	2.340

**Table 5:** Factors associated with satisfaction with the audiovisual.

Variables	Response	Satisfaction		COR(CI)	*p*-Value	AOR(CI)	*p*-Value
		Yes	No				
Age in years	30–39 (ref)	70	104	1		1	
	40–49	76	67	1.69 (1.09–2.64)	0.022	2.199 (1.33–3.63)	0.002 *
	50–59	27	35	1.15 (0.64–2.06)	0.648	1.49 (0.77–2.87)	0.237
	60–69	11	16	1.02 (0.45–2.33)	0.960	1.10 (0.42–2.90)	0.853
Sex	Male (ref)	73	99	1		1	
	Female	111	123	1.22 (0.82–1.82)	0.318	1.447 (0.89–2.37)	0.141
Religion	Orthodox (ref)	101	134	1		1	
	Muslim	53	69	1.02 (0.66–1.59)	0.933	1.131 (0.71–1.81)	0.609
	Other &	30	19	2.10 (1.12–3.93)	0.021	1.916 (0.98–3.74)	0.056
Marital status	Single (ref)	21	18	1		1	
	Married	147	189	0.667 (0.34–1.30)	0.232	0.668 (0.31–1.46)	0.311
	Divorced	6	4	1.286 (0.31–5.28)	0.727	1.167 (0.26–5.32)	0.842
	Widowed	10	11	0.779 (0.27–2.26)	0.646	0.769 (0.22–2.67)	0.679
Educational status	Cannot read and write (ref)	79	111	1		1	
	Can read and write	10	13	1.081 (0.45–1.59)	0.862	1.082 (0.43–2.74)	0.867
	Primary school	30	49	0.860 (0.50–1.74)	0.584	0.990 (0.54–1.80)	0.975
	Secondary school	25	26	1.351 (0.73–2.51)	0.342	1.802 (0.81–4.00)	0.148
	Technical	5	5	1.401 (0.39–5.02)	0.600	2.178 (0.452–10.52)	0.333
	University/higher education	35	18	2.732 (1.44–5.17)	0.002	4.424 (1.36–14.40)	0.014 *
Occupation	Farmer (ref)	57	84	1		1	
	Merchant	33	55	0.884 (0.51–1.53)	0.659	0.682 (0.37–1.26)	0.225
	Private employee	16	19	1.241 (0.59–2.62)	0.570	0.591 (0.21–1.65)	0.316
	Government employee	32	21	2.246 (1.18–4.28)	0.014	0.674 (0.21–2.22)	0.516
	Other ¶	46	43	1.576 (0.92–2.69)	0.095	1.273 (0.68–2.40)	0.454

& (Protestant, Catholic, or Adventist), ¶ (daily laborer or housewife), * (*p*-value < 0.05), COR (crude odds ratio), AOR (adjusted odds ratio), CI (95% confidence interval).

## Data Availability

The data presented in this study are available on request from the corresponding author.

## Supplementary Materials

The following supporting information can be downloaded at: https://www.mdpi.com/article/10.3390/diseases12110294/s1, Materials S1: Health communication materials developed to promote NCD screening in Ethiopia (https://doi.org/10.5281/zenodo.12765170); Table S1: Questionnaire for satisfaction assessment towards the health communication materials on promoting NCD screening among primary health facility attendants; Table S2: Modified clear communication index score sheet.
