# Supplementary material for: The Promotion of Non-Communicable Disease Screening in Gurage Zone, Ethiopia: A Mixed-Method Study
Source: Diseases. 2024 Nov 17;12(11):294. doi: 10.3390/diseases12110294 (PMC11592707; doi:10.3390/diseases12110294)
Supplement: Supplementary file 1 [file diseases-12-00294-s001.zip › Table S1.pdf]

Questionnaire for satisfaction assessment towards the health communication materials on promoting NCD screening among primary health facility attendants

**Section 1: Questions concerning socio-demographic information**		
**No.**	**Questions**	**Categories**
101	Age in years	________
102	Sex	Male---------1<br>Female --------2
103	Religion	Orthodox-------------1<br>Muslim----------------2<br>Protestant-------------3<br>Catholic----------------4<br>Other (specify)--------5
104	Marital status	Single ---------------------1<br>Married -------------------2<br>Divorced ------------------3<br>Widow---------------------4
105	Educational status	Cannot read and write-------1<br>Read and write (without primary school)------2<br>primary school----------3<br>Secondary school ------------4<br>Technical ----------------------5<br>Vocational -----------------------6<br>University / higher education---7
106	Occupation	Farmer---------------------------1<br>Merchant ------------------------2<br>Daily laborer -------------------- 3<br>Private employee ----------------4<br>Government employee----------5<br>Other(specify)--------------------6

**Sec.2: Questions concerning exposure**							
207	Which one you seen/heard	Billboard ------1<br>Audio-visual---2<br>Posters----------3<br>Leaflets--------4					
**Section 3: Questions concerning satisfaction**							
**Level of satisfaction towards audio-visual material**			**Very dissatisfied**	**Dissatisfied**	**Neutral**	**Satisfied**	**Very satisfied**
301	How satisfied are you with the overall presentation of the video						
302	How satisfying is the video in terms of attracting attention						
303	How satisfying did you find its easiness to understand						
304	How did you find appropriateness of the video in terms of culture						
305	How satisfying is the video in terms of giving new/different information						
