# Supplementary material for: The Promotion of Non-Communicable Disease Screening in Gurage Zone, Ethiopia: A Mixed-Method Study
Source: Diseases. 2024 Nov 17;12(11):294. doi: 10.3390/diseases12110294 (PMC11592707; doi:10.3390/diseases12110294)
Supplement: Supplementary file 1 [file diseases-12-00294-s001.zip › Table S2.pdf]

Print Form Reset Form

Modified CDC Clear Communication Index Score Sheet

Name of material ____

Name of person scoring ____

Date __ __ / __ __ / __ __ __ __

NOTE: Use this score sheet for short form and oral communication materials. Examples include Facebook posts, Twitter messages, scripts for podcasts and call center responses, and infographics. The shorter length of social media messages and some oral messages eliminates a few Index items, and oral delivery eliminates the need for information display items.

Before you begin, identify your primary audience, their health literacy skills, your primary communication objective, and main message. You must know these 4 pieces of information to score the material accurately. If you don't have this information, wait until you do to score the material.

Note about translated materials: If the audiences for the English and non-English versions are different, you should create and score the materials separately to account for audience differences.

1. **Who is your primary audience?** ______________________________________

Note: See Appendix B of the User Guide for a list of common public health audiences.

2. **What do you know about the health literacy skills of your audience?**

List as many relevant characteristics about your audience as you can. Try and include evidence about their literacy and numeracy skills; words, numbers, and health concepts they find familiar; their prior experience with the topic; and their ability to comprehend different information formats, such as graphs. <u>If you don't have any information at all, assume average to low health literacy skills.</u>

3. **What is your primary communication objective?**

A communication objective is what you want your audience to think, feel, or do after they receive the message or material. Example 1: Increase the proportion of women between 18-25 years who intend to increase consumption of folic acid. Example 2: Increase the proportion of sexually active adults with favorable attitudes about taking an HIV test.

4. **What is the main message statement in the material?**

The main message statement is the one thing the audience must remember. The statement may be 1-3 short sentences.

If you are reviewing an existing material with multiple messages, list all possible messages.

Revised July 2014

Save Form Next Page

Using the Score Sheet

The Modified Index has a total of 13 items in 4 parts. These 13 items are presented as questions. The number of items you score depends on the type of material.

- Part A **applies to all materials.**
- Parts B, C, and D may not apply to all materials.
- Choose one answer for each item you score.
- Only score a point when all instances of an item in the material meet the criteria.

More detailed descriptions and examples of each item can be found in the User Guide.

**Part A: Core**<br>**Items 2 and 6 have a "not applicable" (NA) option.**	
**Questions**	**Score** *(Check one per question)*
**Main Message and Call to Action**	
**1. Does the material contain one main message statement?**<br>*A main message is the one thing you want to communicate to a person or group that they must remember. A topic, such as heart disease or seasonal flu, isn't a main message statement. If the material contains several messages and no main message, answer no. (User Guide page 5)*<br>**NOTE:** If you answered **No** to Question 1, **score 0 for Question 2** and **continue** to Question 3.	☐ Yes = 1<br>☐ No = 0
**2. Is the main message at the top, beginning, or front of the material?**<br>*The main message must be in the first paragraph or section. A section is a block of text between headings. (User Guide page 6)*<br>**NOTE:** This item isn't applicable to 1-3 sentence messages, such as tweets.	☐ Yes = 1<br>☐ No = 0<br>☐ NA
**3. Does the material include one or more calls to action for the primary audience?**<br>*If the material includes a specific behavioral recommendation, a prompt to get more information, a request to share information with someone else, or a broad call for program or policy change, answer yes. If the call to action is for someone other than the primary audience, answer no. (User Guide page 10)*	☐ Yes = 1<br>☐ No = 0
**Language**	

Save Form   Next Page

Previous Page

4. **Do both the main message and the call to action use the active voice?**<br>*If only the main message or only the call to action uses the active voice, answer no. If you answered no to #1 or #3, answer no. (User Guide page 11)*	☐ Yes = 1<br>☐ No = 0
5. **Does the material always use words the primary audience uses?**<br>*If all specialized or unfamiliar terms are explained or described (not just defined) the first time they are used, answer yes. Acronyms and abbreviations must be spelled out and explained if unfamiliar to the audience. (User Guide page 12)*	☐ Yes = 1<br>☐ No = 0
6. **Is the most important information the primary audience needs summarized in the first paragraph or section?**<br>*The most important information must include the main message. A section is a block of text between headings. (User Guide page 17)*<br>**NOTE:** This item isn't applicable to 1-3 sentence messages, such as tweets, and infographics.	☐ Yes = 1<br>☐ No = 0<br>☐ NA

Part A score **Total ______ / 6**

Comments

Save Form

Next Page

Revised July 2014

Part B: Behavioral Recommendations

Answer this question to determine if items 7and 8 apply to the material.

Does the material include one or more behavioral recommendations for the primary audience?

- If **yes** – score items 7 and 8.
- Item 8 has a “not applicable” (NA) option.
- If **no** – skip to Part C. Go to Part C

Questions	Score (Check one per question)
7. **Does the material include one or more behavioral recommendations for the primary audience?**<br>*If no, STOP here and don't score Part B. (User Guide page 19)*	☐ Yes = 1
8. **Does the material explain why the behavioral recommendation(s) is important to the primary audience?**<br>*If you offer only numbers to explain the importance of the behavioral recommendation with no other relevant information for the audience, answer no. (User Guide page 20)*<br><br>**NOTE:** This item isn't applicable to 1-3 sentence messages, such as tweets.	☐ Yes = 1<br>☐ No = 0<br>☐ NA

Part B score **Total ______ / 2**

Comments

Save Form Next Page

Previous Page

Part C: Numbers

Answer this question to determine if items 9 and 10 apply to the material.

Does the material include one or more numbers related to the topic?

- If **yes** – score items 9 and 10.
- If **no** – skip to Part D. Go to Part D

Questions	Score (Check one per question)
**9. Does the material <u>always</u> present numbers the primary audience uses?** *Many audiences find numbers distracting or confusing. Make sure the numbers in the material are both familiar and necessary to support or explain the main message statement. If not, delete the numbers. Whole numbers are used by most audiences. The types of numbers used will vary for each audience. (User Guide page 22)*	☐ Yes = 1<br>☐ No = 0
**10. Does the audience have to conduct mathematical calculations?** *Adding, subtracting, multiplying, and dividing involve calculations. Calculating a common denominator for the purposes of comparison is a mathematical calculation. Use the same denominator, even for absolute risk (example: 1 out of 3), throughout the material so that audiences don't have to calculate. (User Guide page 24*<br><br>**NOTE: for this item, Yes is scored 0 and No is scored 1.**	☐ Yes = 0<br>☐ No = 1

Part C score **Total ______ / 2**

Comments

Save Form Next Page

Previous Page

Modified CDC Clear Communication Index Score Sheet

Part D: Risk

Answer this question to determine if items 11-13 apply to the material.

Does the material present information, including numbers, about risk?

- If **yes** – score items 11-13.
- Items 12 and 13 have a "not applicable" (NA) option.
- If **no** – skip to Calculate the Score. Go to Calculate

Questions	Score (Check One per Question)
**11. Does the material explain the nature of the risk?**<br>*If the material states the threat or harm and how and why people may be affected, answer yes. If the material has only the threat or harm but no explanation, answer no. For example, if the material states there are 1,000 new cases of a contagious disease in Springfield, does it also state that people in Springfield may be more likely to get the disease, why they may be more likely, and how serious the threat of the disease is? (User Guide page 26)*	☐ Yes = 1<br>☐ No = 0
**12. Does the material address both the risks and benefits of the recommended behaviors?**<br>*This includes actual risks and benefits and those perceived by your audience. If the material addresses only risks or only benefits, answer no. If no behavioral recommendation is presented, answer not applicable (NA). (User Guide page 27)*<br>**NOTE:** This item isn't applicable to 1-3 sentence messages, such as tweets.	☐ Yes = 1<br>☐ No = 0<br>☐ NA
**13. If the material uses numeric probability to describe risk, is the probability also explained with words or a visual?**<br>*Examples of probability information in a risk message are numbers (such as 1 in 5 or 20%). If the material presents numeric risk and also uses text to explain the probability, answer yes. If the material presents numeric risk and also uses a visual to explain the probability, answer yes. If the material only presents numeric risk, answer no. If the material does not include this type of probability information, answer not applicable (NA). (User Guide page 28)*<br>**NOTE:** This item isn't applicable to 1-3 sentence messages, such as tweets.	☐ Yes = 1<br>☐ No = 0<br>☐ NA

Part D score Total ____ / 3

Save Form

Next Page

Previous Page

Calculate the Score for the Material

- **Step 1:** The total points that the material earned (this is the numerator).
  - A: _____ B: _____ C: _____ D: _____ = _____
- **Step 2:** The total possible points that the material could have earned (this is the denominator).
  - A: _____ B: _____ C: _____ D: _____ = _____
  - Maximum possible points for online posts 2 paragraphs or less, and scripts for audio recordings and prepared responses: 11, 12 or 13 depending on the number of items scored in Part D
  - Maximum possible points for infographics: 10, 11 or 12 depending on the number of items scored in Part D
  - Maximum possible points for 1-3 sentence messages: 8
- **Step 3:** The numerator divided by the denominator multiplied by 100 to get the total score.

______ / ______ x 100 = ______

How to Interpret the Score

The purpose of the Index is to improve the clarity of communication products.

If the total score is 90 or above:

Excellent! You have addressed most items that make materials easier to understand and use.

If the total score is 89 or below:

Note which items scored 0 points. Use the descriptions and examples in the User Guide to revise and improve the material. Then apply the Index again to check your work. You can use the Index as many times as you need to revise the material to get a score of 90 or above.

Additional Comments

Email Form Print Form Save Form
